# An Investigation on the Solid Dispersions of Chlordiazepoxide

**Published:** 2007-09

**Authors:** Ali Nokhodchi, Roya Talari, Hadi Valizadeh, Mohammad Barzegar Jalali

**Affiliations:** 1 *Medway School of Pharmacy, the Universities of Kent and Greenwich, Central Ave., Chatham Maritime, ME4 4TB, Kent, UK;*; 2 *School of Pharmacy, Zanjan University of Medical Sciences, Zanjan, Iran;*; 3 *Drug Applied Research Center and School of Pharmacy, Tabriz University of Medical Sciences, Tabriz, Iran*

**Keywords:** chlorodiazepoxide, solid dispersion, dissolution rate, cogrinding

## Abstract

The aim of this study was to prepare solid dispersions of chlordiazepoxide techniques to improve its dissolution rate. To this end, three techniques namely, two solvent methods and co-grinding technique were used. Solid dispersions of chlordiazepoxide in polyvinylpyrrolidone (PVP), Eudragit E100, Mannitol and Sorbitol with two different ratios of drug to carrier (5:5 and 1:9) were prepared. These solid dispersions were evaluated using dissolution tester to monitor dissolution behaviour and Fourier-transform infrared spectroscopy to investigate interaction between the drug and carriers in solid dispersion samples. Solid dispersion of chlordiazepoxide with all three carriers (PVP, mannitol and eudragit E) prepared by solvent method showed considerable increase in the dissolution rate of chlordiazepoxide in comparison with physical mixture and pure drug at different pH values. According to the results of this investigation cogrinding technique yields solid dispersions with a less improved dissolution rate than does the solvent deposition technique. Infrared studies showed no interaction between chlordiazepoxide and carriers in solid dispersions in solid state.

## INTRODUCTION

Many potential drug candidates are characterized by a low oral bioavailability. Often, poor drug dissolution and solubility rather than limited permeation through the epithelia of the gastrointestinal tract are responsible for low bioavailability of orally taken drugs. Therefore, together with permeability, the solubility and dissolution behavior of a drug are key determinants of its bioavailability when administered orally. Among the techniques to increase aqueous solubility/dissolution rate, the formulation of solid dispersions is one of the most popular ones (1-4), although few marketed products rely on this concept. This approach frequently improves bioavailability that is limited or rate-controlled by dissolution (5). Different polymers and sugars have frequently been used as carrier in solid dispersion formulations (6-11). Mechanisms suggested to be responsible for the improved aqueous solubility/dissolution properties of solid dispersions include reduction of the particle size of the incorporated drug, partial transformation of the crystalline drug to the amorphous state, formation of solid solutions, formation of complexes, reduction of aggregation and agglomeration, improved wetting of the drug and solubilisation of the drug by the carrier at the diffusion layer (2, 12-14). It is highly acceptable, that often more than one of these phenomena determine the rate and extent of dissolution. It has been shown that the methods for the preparation of solid dispersions are simple and less expensive than other techniques (7, 8, 11, 14, 16-18).

Chlordiazepoxide, a member of the 1, 4-benzodiazepine group, showing poor aqueous solubility and dissolution rate in water, was selected as model drug. The drug is a classical example of a solubility problem wherein it exhibits a 4000-fold difference in solubility going from pH 3 to 6 (the solubility of chlordiazepoxide hydrochloride is 150 mg/ml and is ~0.1 mg/ml at neutral pH) (19). Patients with hypochloridia or achloridia usually have higher stomach pH which causes a reduction in the solubility of chlordiazepoxide hence a significant reduction in the dissolution rate of chlordiazepoxide samples. Therefore, the present investigation was focused on exploring PVP, mannitol and eudragit E100 as carriers to increase the dissolution rate of chlordiazepoxide by formation of solid dispersions. Also studied was the effect of solid dispersion technique (solvent evaporation, co-precipitation and co-grinding techniques) on dissolution of chlordiazepoxide.

## EXPERIMENTAL

### Materials

Chlordiazepoxide and Eudragit E100 were purchased from F.I.S., Italy and Rohm Pharma, Germany, respectively. Polyvinylpyrrolidone K30 (PVP), mannitol, sorbitol and hydrochloric acid were obtained from Merck (Germany).

### Preparation of physical mixtures

Physical mixtures of chlordiazepoxide (<250 μm) were prepared by mixing accurately weighed amounts of chlordiazepoxide with carriers (ratio of drug: carrier was 5:5 and 1:9) in geometric proportions for 10 min in a blender (Golden stars, French) at a speed of 20 rpm, until a homogenous mixture was obtained. The physical mixtures were subsequently stored at room temperature in a screw-capped glass vial until use.

### Preparations of solid dispersions

Solid dispersions with different concentrations of chlordiazepoxide were prepared using the following three techniques:

**Solid dispersions prepared by the solvent evaporation method.** Solid dispersions containing 10 and 50% w/w of chlorodiazepoxide was prepared by dissolving accurately weighed amounts of PVP K30 and drug in ethanol. After complete dissolution, the solvent was evaporated under reduced pressure at 60°C in a desiccator. Subsequently, the solid mass was ground and the particle size fraction of <250 μm was obtained by sieving. The sieved ground powders were stored in an oven for at least 48 h. All dispersions were kept at room temperature in a screw-capped glass vial until use. Similar procedure was carried out to prepare chlordiazpoxid solid dispersions containing eudragit E.

**Solid dispersions prepared by the co-precipitation method (coevaporates).** Solid dispersions containing 10 and 50% w/w of chlorodiazepoxide were prepared by dissolving accurately weighed amounts of mannitol in water and drug in ethanol. After complete dissolution, the aqueous solution of carrier was then poured into the ethanolic solution of the drug. The solvents were then heated and evaporated under reduced pressure at 60°C in a dessicator. Subsequently, the solid dispersions were stored in an oven for 48 h at 60°C. All dispersions were pulverized with pestle and mortar, sieved (<250 μm) and dried in an oven for at least 48 h. All dispersions were kept at room temperature in a screw-capped glass vial until use.

**Solid dispersions prepared by co-grinding method.** Pure chlordiazepoxide powder and the carrier (PVP or mannitol) were physically mixed for 10 min using a blender at a speed of 20 rpm. The mixture was then charged into the chamber of a vibration ball mill (Fritsch, Utzenhofen, Germany). A certain number of steel balls were added in a way that the total volume of powder mixture and balls equaled one-third the volume of the ball mill chamber. The powder mixtures were ground for 1 h at a vibration speed 360 rpm. Then, the samples were collected and kept at room temperature in a screw-capped glass vial until use.

### Infrared spectroscopy

Fourier-transform infrared spectroscopy (FT-IR) was obtained on a Bomem 2000 FT-IR system (Bomem Qubec, Canada) using the KBr disk method. Samples were mixed with KBr powder and compressed to 10-mm discs by hydraulic press at pressure of 10 tons for 30 s. The scanning range was 500-4000 cm^-1^ and resolution was 2 cm^-1^.

### Dissolution studies

Dissolution studies were carried out using the paddle method (USP XXIV, apparatus II). Samples of drug, solid dispersions and physical mixtures equivalent with 10 mg drug were immersed in the dissolution medium consisted of 900 ml simulated gastric fluid (HCl, pH 1.2 or 3) without pepsin which was maintained at 37°C. The duration of the test and the rate of paddle stirring were 90 min and 100 rpm, respectively. At designated time intervals, 5 ml samples were withdrawn, and replaced with 5 ml of fresh dissolution medium. The samples were filtered through a filter paper and analyzed by UV spectrophotometry at 246 nm. Cumulative percentages of the drug dissolved from the preparations were calculated. The dissolution experiments were performed in triplicate for each sample. In order to investigate the behaviour of samples in distilled water, the dissolution test was also carried out in distilled water as dissolution medium.

## RESULTS AND DISCUSSION

**Figure 1:**
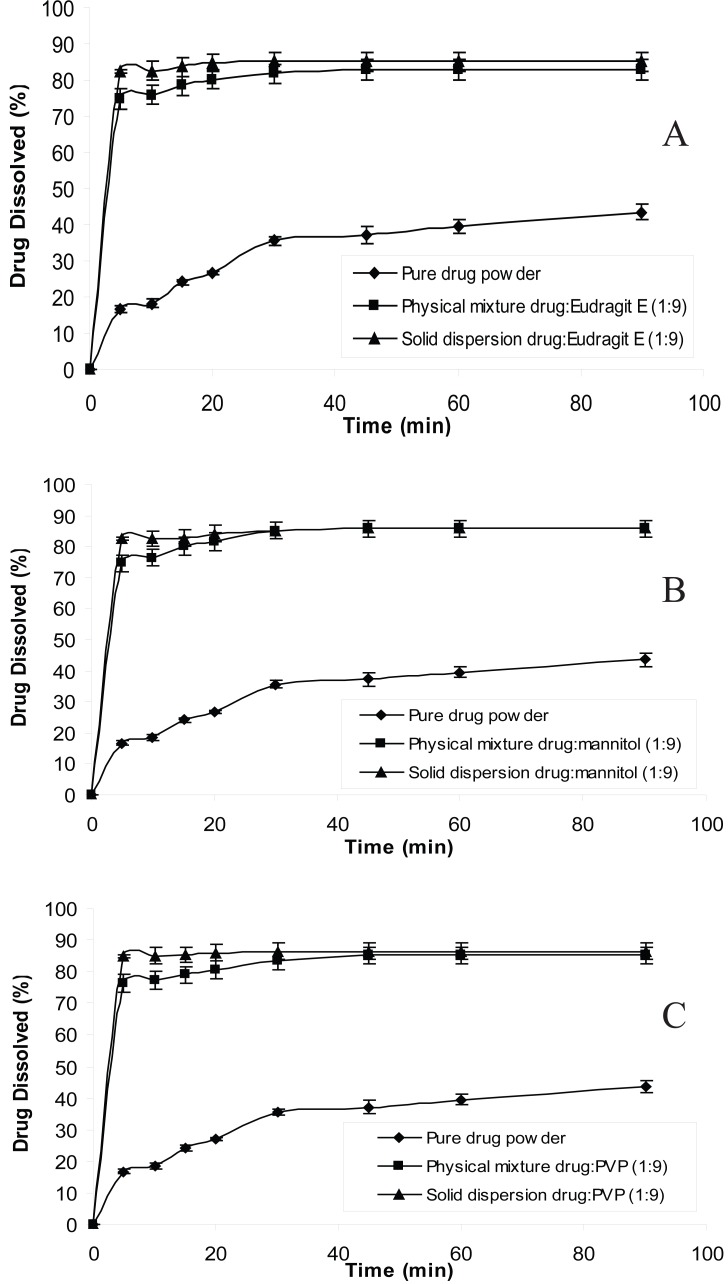
Dissolution profiles of pure chlordiazepoxide, solid dispersions and physical mixtures of drug with different carriers at pH 1.2 (ratio of drug:carrier is 1:9).

**Figure 2:**
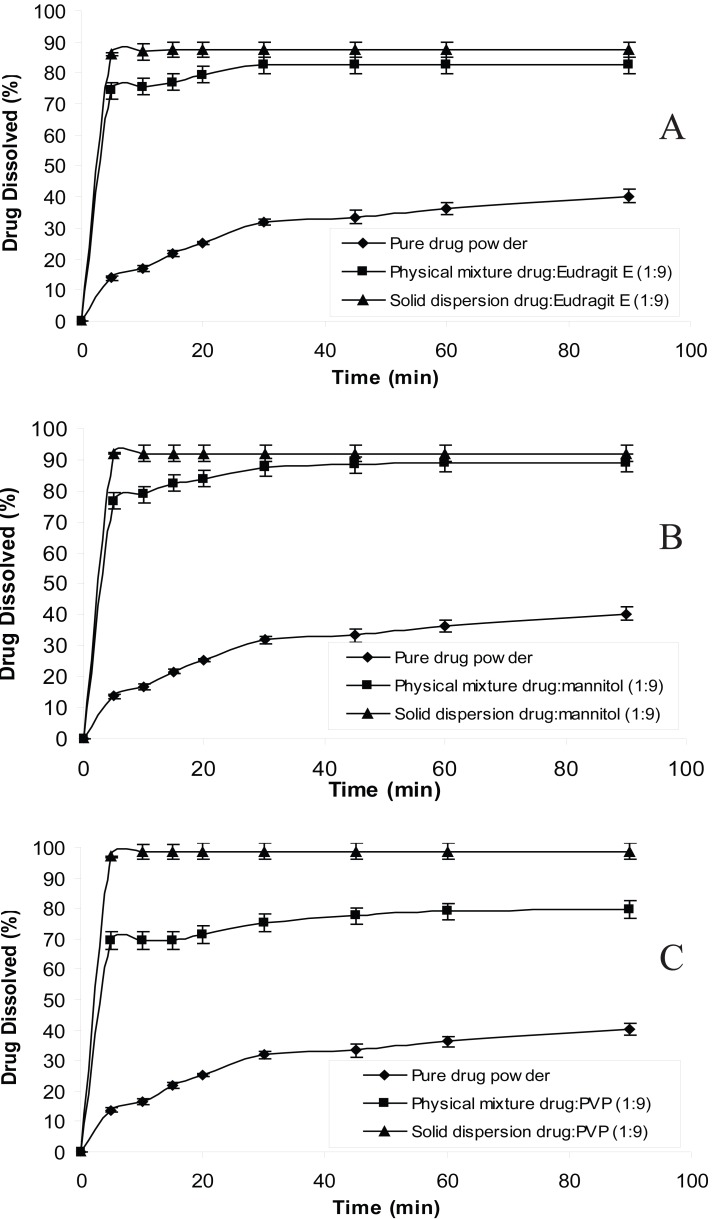
Dissolution profiles of pure chlordiazepoxide, solid dispersions and physical mixtures of drug with different carriers at pH 3 (ratio of drug:carrier is 1:9).

**Figure 3:**
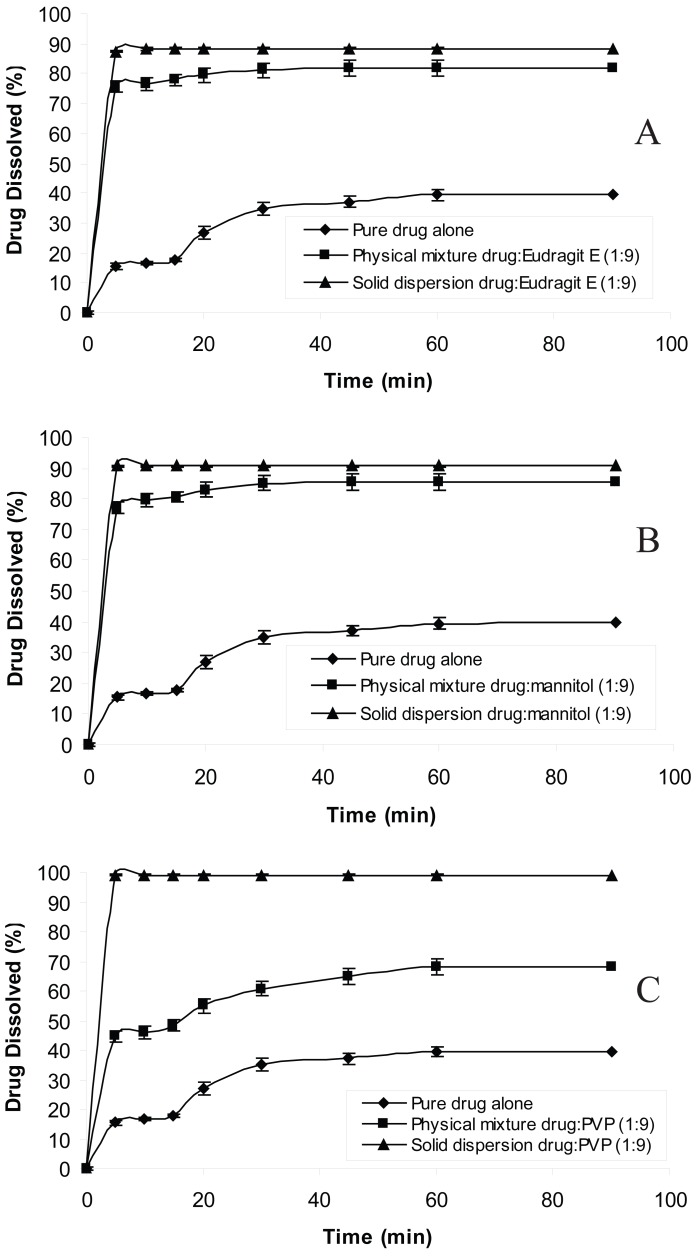
Dissolution profiles of pure chlordiazepoxide, solid dispersions and physical mixtures of drug with different carriers in distilled water (ratio of drug:carrier is 1:9).

The dissolution behavior of chlordiazepoxide from solid dispersions and physical mixtures was analyzed in simulated gastric fluid without pepsin (pH 1.2 and 3). The dissolution test was also carried out in distilled water according to USP 26 requirement. The dissolution profiles of all formulations at pH 1.2, 3 and distilled water are shown in Figures 1, 2 and 3, respectively. From the figures it is evident that the dissolution of pure chlordiazepoxide at different pH values is very low. For example, only 16, 14 and 15% of the drug is dissolved within 5 min at pH 1.2, 3 and distilled water, respectively. In the case of physical mixtures, co-habitation of carriers with chlordiazepoxide clearly improved the dissolution rate of the drug when the ratio of drug:carrier was 1:9. These figures clearly show that the dissolution rate of all physical mixtures is higher than the pure chlordiazepoxide.

Consideration of the modified Noyes-Whitney equation (20) provides some hints as to how the dissolution rate of poorly water-soluble drugs might be improved to minimize the limitations to oral availability:

dc/dt = [AD(C_S_-C)/h]

where dc/dt is the rate of dissolution, A is the surface area available for dissolution, D is the diffusion coefficient of the drug, C_S_ is the solubility of the drug in the dissolution medium, C is the concentration of drug in the medium at time t and h is the thickness of the diffusion boundary layer adjacent to the surface of the dissolving drug. The main possibilities for improving dissolution according to this analysis are to be increase the surface area available for dissolution by decreasing the particle size of the solid compound and/or by optimizing the wetting characteristics of the compound surface, to decrease the boundary layer thickness, to improve the apparent solubility of the drug.

It is clear from Figures 1 to 3 that all solid dispersion formulations produced higher dissolution rate than the physical mixtures and the pure drug. Formulation of solid dispersions is expected to further improve the dissolution behaviour of chlordiazepoxide in comparison with the physical mixtures by reducing the drug particle size, formation of drug-carrier solid solutions, and transformation of the drug into a faster dissolving amorphous state and by a more intimate contact between the carrier and the drug. The influence of carriers on the dissolution of chlordiazepoxide can be explained by formation of regions containing high concentration of dissolved carrier at the surface of drug crystals in which the drug can be solubilised. This will improve the subsequent diffusion of the drug followed by dilution in the bulk of the solution. While a number of theories have been proposed the mechanism by which the dissolution rate is improved in relation to physical mixtures is not fully understood. The results indicate that the selected carriers (eudragit E, mannitol and PVP) are suitable carriers for increasing the dissolution rate of chlordiazepoxide solid dispersion at various pH values. The comparison of dissolution profiles of solid dispersions of chlordiazepoxide containing different carriers revealed that PVP is the superior carrier, improving the dissolution rate of chlordiazepoxide more efficiently than the other carriers. However, it must be noted that the difference between PVP and mannitol is not supported by statistical analysis (*p*>0.05 in ANOVA test).

As all these carriers are hydrophilic, an increase in the amount of carriers can also enhance the hydrophilicity of the surface of the solid dispersion particles and this, in turn, reduces the agglomeration of drug particles after exposure to the dissolution medium (21).

**Figure 4:**
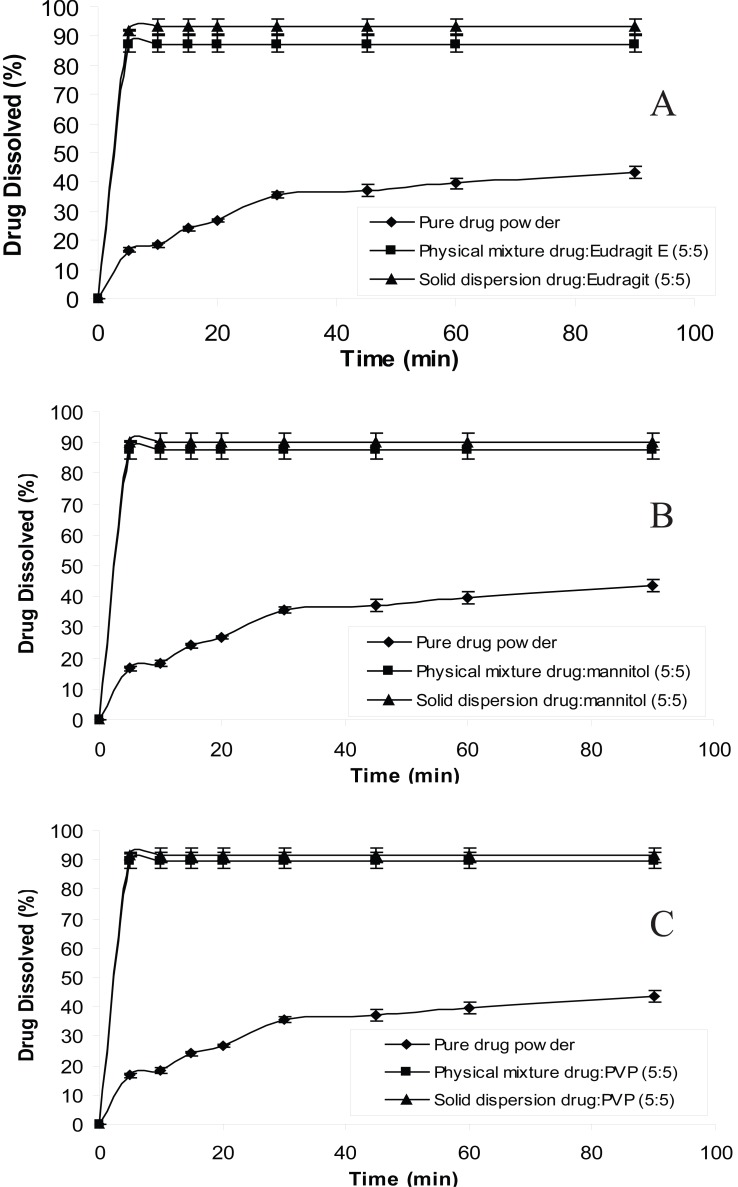
Dissolution profiles of pure chlordiazepoxide, solid dispersions and physical mixtures of drug with different carriers in distilled water (ratio of drug:carrier is 5:5).

In order to reduce the amount of carrier used in solid dispersion formulations the ratio of drug:carrier was increased from 1:9 to 5:5 and dissolution studies were carried out at distilled water. The reason for choosing distilled water as the dissolution medium is that chlordiazepoxide has a low solubility in water than in acidic media; solubility of chlordiazepoxide at pH 3 and distilled water are 150 mg/ml and 0.1 mg/ml, respectively (19). Therefore, the dissolution data for 5:5 ratio of drug:carrier is plotted in Figure 4. The figure shows that even in this ratio solid dispersions produced higher dissolution rate than the physical mixtures and the drug alone. The drug/carrier ratio in a solid dispersion is one of the main factors affecting the performance of a solid dispersion. It has been shown that if the percentage of the drug is too high (high ratio of drug/carrier), it would form small crystals within the dispersion rather than remaining molecularly dispersed. On the other hand, if the percentage of the carrier is very high (low ratio of drug/carrier), this could lead to the complete absence of crystallinity of the drug and thereby enormous increases in the solubility and dissolution rate of the drug (22). Therefore, dissolution profile of 5:5 ratio of drug:carrier shows that the drug is probably molecularly dispersed at this ratio and crystallization of drug has not occurred.

Although the results showed that the physical mixtures of chlordiazepoxide and carrier have very good dissolution rate at pH 1.2, solid dispersion can have better therapeutic effect for patients with hypochloridia or achloridia. A study (23) showed that in a group of 100 normal people over the age of 60, over 50% had hypochloridia (this simply means that they did not make enough hydrochloric acid). Furthermore, worse still was the fact that this and other studies reveal that many people over the age of 65 make no HCl at all (known as achloridia). The solid dispersion produced in this investigation improved the dissolution rate at pH 3 and distilled water (neutral pH) in comparison with physical mixtures. Thus, the solid dispersions of chlordiazepoxide can be ideal formulations for this type of patients. Moreover, the solid dispersion will benefit the bioavailability of the drug when taken with food, as presence of food normally increases the pH of the stomach potentially reducing the solubility and bioavailability of chlordiazepoxide. More explanation was included to clarify the therapeutic benefits of solid dispersions of chlordiazepoxide.

**Figure 5:**
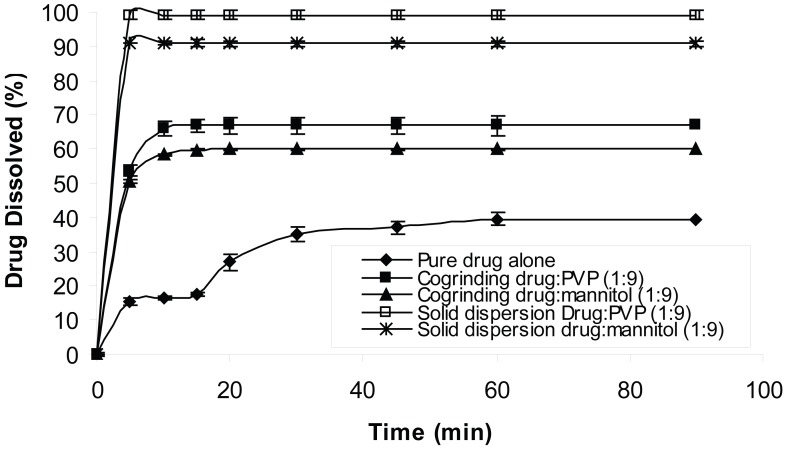
The effect of solid dispersion technique on the dissolution profiles of chlordiazepoxide at distilled water.

In order to compare the effect of solid dispersion technique on dissolution of chlordiazepoxide, cogrinding technique was compared with solvent deposition technique (Fig. 5). The results revealed that the solid dispersions produced by solvent deposition technique have higher dissolution rate than the samples produced by cogrinding technique (Fig. 5). This could be due to molecular dispersion of the drug in the samples when solvent deposition technique was used. It has been shown that cogrinding technique is able to increase dissolution of poorly water-soluble drugs (22, 24). The present study showed the solvent deposition technique is more efficient than cogrinding technique to increase the dissolution of chlordiazepoxide.

**Figure 6:**
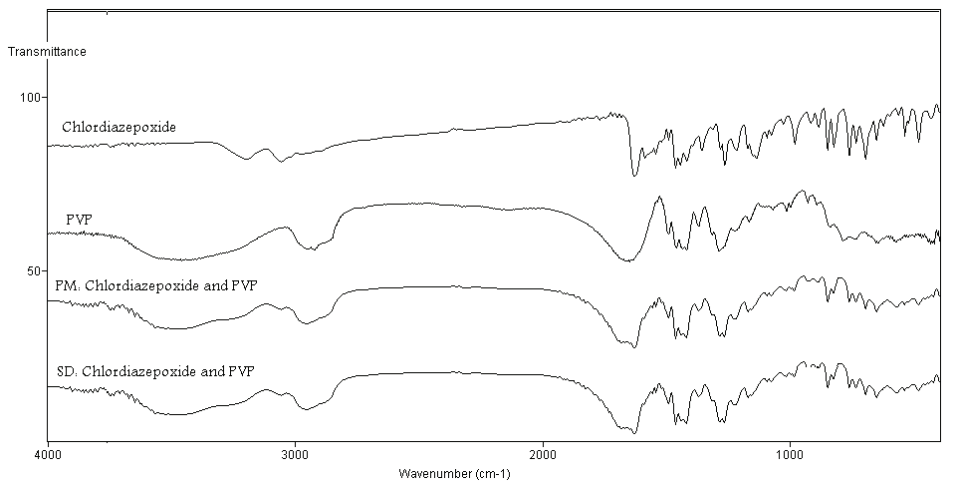
FT-IR spectra pure chlordiazepoxide, PVP, physical mixture (PM) of chlordiazepoxide-PVP and solid dispersion (SD) of chlordiazepoxide-PVP prepared by solvent method (ratio of drug : carrier was 1:9).

In order to study possible interaction between carriers and chlordiazepoxide in the solid state, FT-IR spectra were recorded. Figure 6 shows the FT-IR of physical mixture and solid dispersion samples prepared by the solvent evaporation technique containing chlordiazepoxide and PVP. From the chemical structures, hydrogen bonding could be expected between PVP and chlordiazepoxide. These interactions would result in peak broadening. Comparing the spectra of physical mixtures with the solid dispersions sample prepared by the solvent evaporation technique ruled out differences in the positions of the absorption bands for chlordiazepoxide in these samples, hence providing evidence for the absence of hydrogen bonding interactions in the solid state between PVP and chlorodiazepoxide (Fig. 6). Similar results were obtained for other solid dispersion samples containing mannitol or eudragit E (FT-IR spectra were not shown here).

## CONCLUSION

This study clearly shows that addition of carriers especially PVP to chlordiazepoxide improves its dissolution rates. In some of formulations solid dispersions did not further improve the dissolution rate compared with physical mixtures. The present study also showed that cogrinding technique yielded solid dispersions with a less improved dissolution rate than did the solvent deposition/evaporation technique. Results from FT-IR spectroscopy concluded that there was no well-defined interaction between chlordiazepoxide and PVP, mannitol or eudragit E100.
